# Primary Tracheal Leiomyoma in a Dog: A Case Report

**DOI:** 10.3390/ani16172810

**Published:** 2026-09-07

**Authors:** María Ezquerra-Durán, María Esther Durán-Flórez, Luis Javier Ezquerra-Calvo, Pablo Cardenal-Morales, Massimo Santella

**Affiliations:** 1 Small Animal Surgery Unit, Veterinary Teaching Hospital, Universidad de Extremadura, 10003 Caceres, Spain; mariaezquerra4@gmail.com (M.E.-D.); msantella@unex.es (M.S.); 2 Pathology Unit, Animal Medicine Department, Universidad de Extremadura, 10003 Caceres, Spain; esther@unex.es; 3 Surgery Unit, Animal Medicine Department, Universidad de Extremadura, 10003 Caceres, Spain; 4 MINVET Research Group, Animal Medicine Department, Universidad de Extremadura, 10003 Caceres, Spain; pablocm@unex.es

**Keywords:** leiomyoma, trachea, neoplasia, canine, end-to-end anastomosis

## Abstract

Primary tracheal neoplasia is uncommon in domestic animals and humans, and most reported tracheal tumours are malignant. To the authors’ knowledge, this case represents the third confirmed case of tracheal leiomyoma reported in dogs. In human medicine, tracheal leiomyomas have also only been reported sporadically and account for approximately 1% of tracheal tumours. A 12-year-old spayed female Pit Bull was presented as an emergency because of an acute worsening of recurrent respiratory signs that were refractory to medical treatment. On initial evaluation, reduced mobility of both arytenoid cartilages was observed, and left arytenoid lateralisation was therefore performed. However, four days after the procedure, the dog continued to experience severe episodes of respiratory distress requiring orotracheal intubation. Subsequent bronchoscopy revealed an intraluminal tracheal mass occupying approximately 90% of the airway lumen. Because of this obstruction, an emergency temporary tracheostomy was performed caudal to the mass. Once the dog had been clinically stabilised, computed tomography was performed for surgical planning. Surgical treatment consisted of segmental tracheal resection and end-to-end anastomosis, with removal of a segment corresponding to 24% of the total tracheal length. Histopathological examination confirmed the diagnosis of tracheal leiomyoma. Complete surgical excision was achieved, supporting a favourable prognosis.

## Simple Summary

Primary tracheal neoplasms are uncommon in both domestic animals and humans, and more than 90% of reported tracheal tumours are malignant. To the authors’ knowledge, this is the third confirmed case of tracheal leiomyoma reported in a dog. In human medicine, tracheal leiomyomas have also only been reported sporadically and account for approximately 1% of tracheal tumours. This case report describes a geriatric spayed female Pit Bull with recurrent respiratory signs refractory to medical treatment. The dog was presented on an emergency basis owing to worsening respiratory distress. A tracheal mass partially occluding the airway lumen was identified. Tracheal resection and end-to-end anastomosis were performed, with removal of 24% of the total tracheal length. Histopathological examination confirmed a diagnosis of tracheal leiomyoma.

## 1. Introduction

The incidence of primary tracheal neoplasms in dogs and cats has not been established, but it is considered to be very low, as is also the case in humans, in whom an incidence of 0.142–0.27 cases per 100,000 people has been reported [1,2]. Reported tracheal neoplasms include adenocarcinoma, carcinoma, extramedullary plasmacytoma, leiomyoma, fibrosarcoma, mast cell tumour, rhabdomyosarcoma, and squamous cell carcinoma [3,4,5,6,7,8]. However, most reported tracheal tumours are malignant [9,10].

Regardless of their histological type or biological behaviour, the clinical signs and radiographic findings are usually similar. These tumours tend to cause airway obstruction [11], resulting in non-specific clinical signs, including coughing, dyspnoea, blood-tinged sputum, bronchitis, and aphonia [12,13]. Radiographically, they usually appear as solitary, well-defined intratracheal masses [5].

Tracheal neoplasms are usually reported in middle-aged or older animals, although benign osteochondroma and osteochondral dysplasia have been described in dogs younger than 2 years of age [14,15].

Although only isolated cases have been reported in both human and veterinary medicine, tracheal leiomyomas have been reported more frequently in men than in women [16].

Given the potential for tracheal masses to cause severe, potentially life-threatening airway obstruction requiring prompt intervention, computed tomography (CT) and bronchoscopy are valuable complementary diagnostic modalities for accurately characterising the location and extent of the lesion, assessing luminal involvement and extraluminal extension, obtaining tissue samples, guiding surgical planning, and supporting prognostic assessment [14,17,18]. Depending on the attachment site of the tumour, alternative approaches such as endoscopic resection using a polypectomy snare or laser therapy may be considered [19]. Tracheal resection and anastomosis may provide a longer disease-free interval than endoscopic debulking, even when complete surgical margins are not achieved [20].

## 2. Case Description

### 2.1. Clinical Presentation

A 12-year-old spayed female Pit Bull weighing 27 kg was presented with a history of chronic respiratory signs, including coughing, sneezing, cyanosis, and tachypnoea, characterised by seasonal worsening during the winter months and progressive deterioration over the preceding month. The dog was admitted as an emergency because of signs compatible with severe upper airway obstruction, including inspiratory dyspnoea, tachypnoea, and cyanotic episodes. Examination of the upper airway revealed bilateral laryngeal paralysis (LP), and left arytenoid lateralisation was performed. Haematological and serum biochemical results, including the serum total thyroxine concentration, were within their respective reference intervals. Despite surgical treatment, the dog continued to show signs of respiratory obstruction requiring orotracheal intubation. Emergency bronchoscopy was subsequently performed and revealed an intraluminal tracheal mass causing approximately 90% obstruction of the airway lumen. Because of the severity of the obstruction and the need to secure the airway, a temporary tracheostomy was performed caudal to the mass.

### 2.2. Imaging Diagnosis

**Figure 1:**
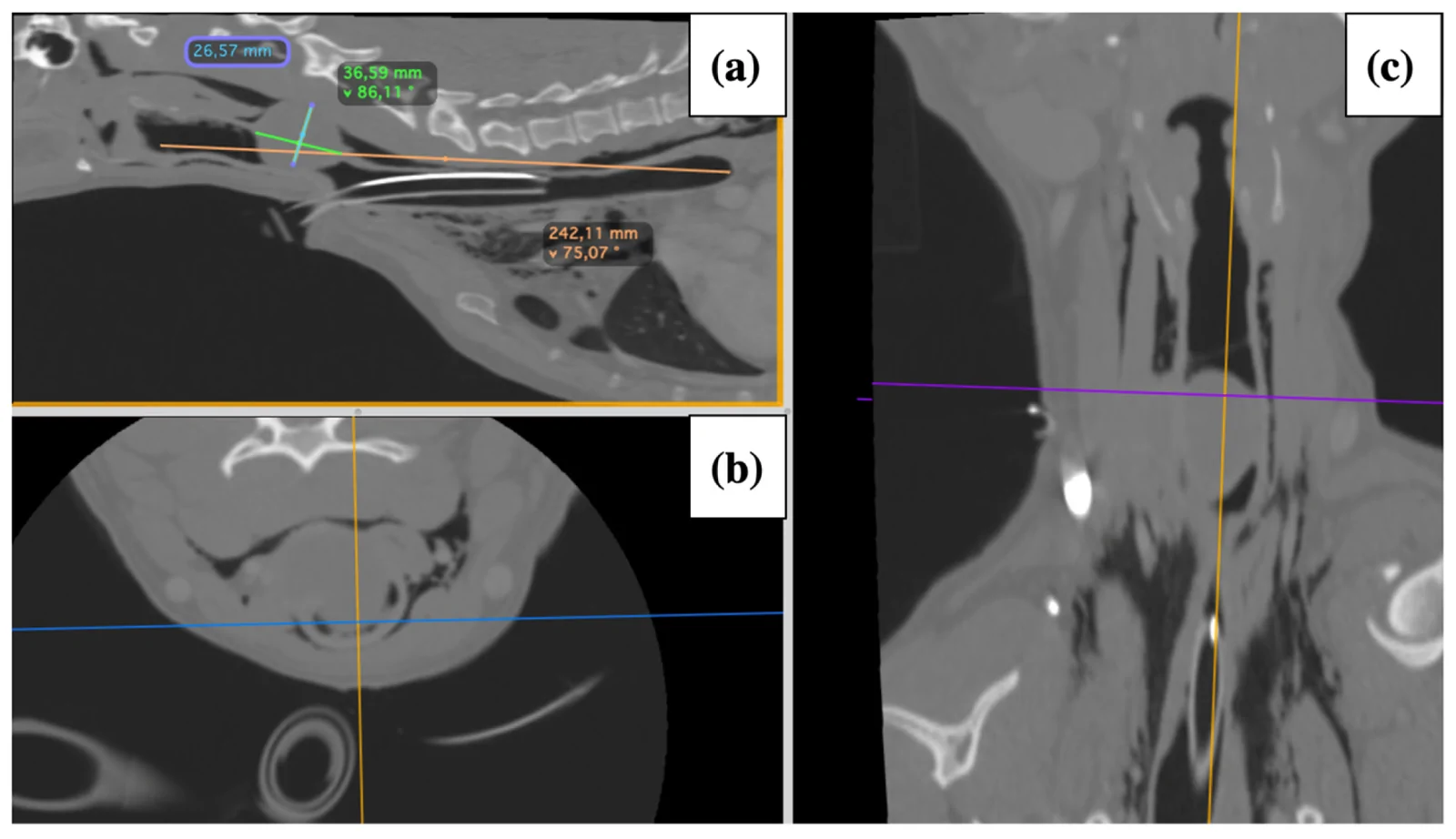
Computed tomography images showing a tracheal mass extending from the ninth to the 15th tracheal ring and occupying approximately 90% of the tracheal lumen: (**a**) sagittal, (**b**) transverse, and (**c**) dorsal planes.

An unenhanced and contrast-enhanced CT examination was performed to plan tumour resection and to assess the presence of additional masses or metastatic disease. CT revealed a tracheal mass extending from the ninth to the 15th tracheal ring and occupying approximately 90% of the tracheal lumen. On unenhanced CT, the mass showed a mean attenuation value of 33 HU, consistent with a soft-tissue lesion. Persistent airway obstruction was observed despite adequate glottic opening following the previous left arytenoid lateralisation. The mass appeared to arise from the dorsal tracheal ligament and caused oesophageal compression at the level of the C3–C4 vertebral bodies (Figure 1). No evidence of metastatic disease was detected.

Thoracic and cervical radiographs did not reveal evidence of the intraluminal tracheal mass, probably because of its low radiographic conspicuity and superimposition of adjacent soft-tissue structures.

### 2.3. Surgical and Anaesthetic Management

**Figure 2:**
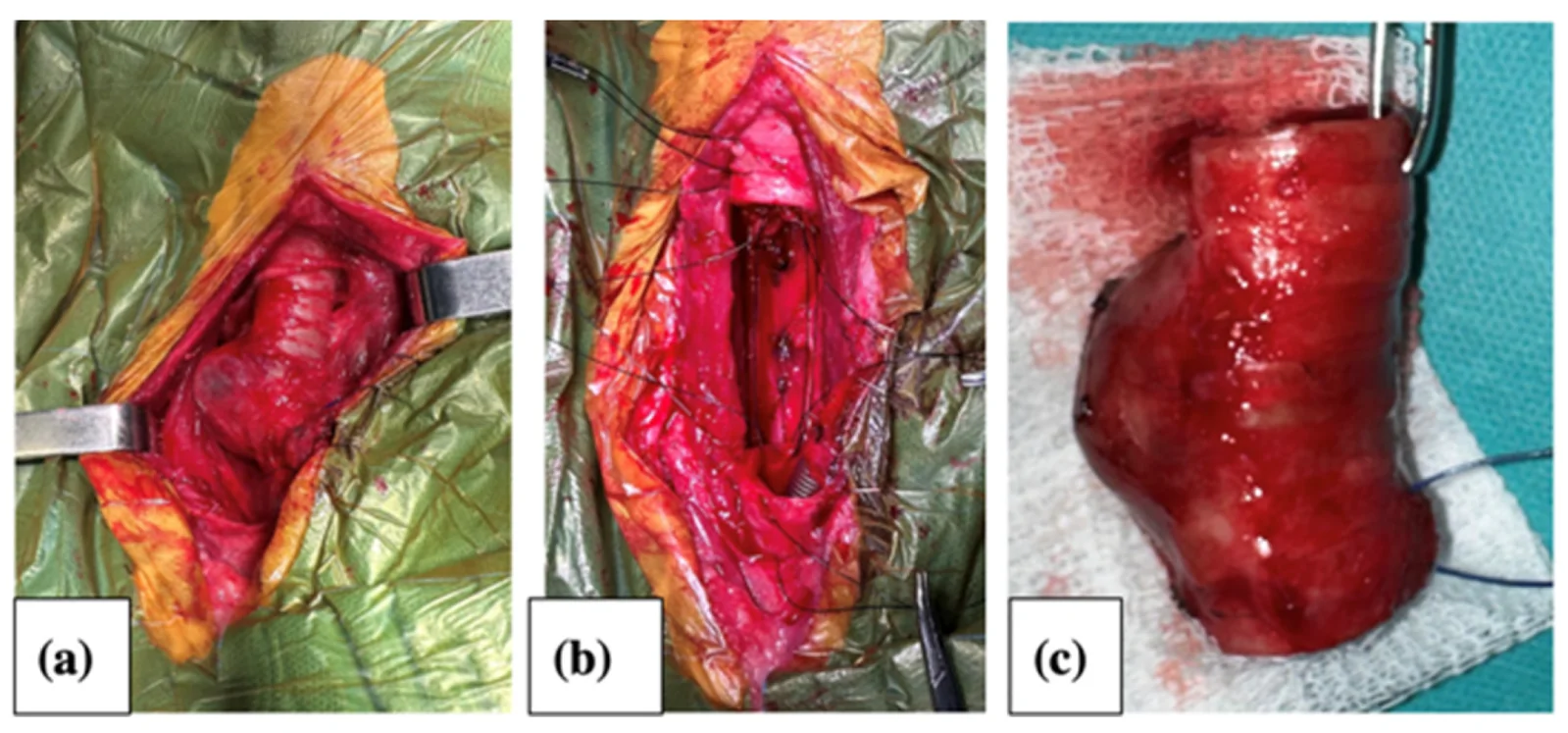
Intraoperative photographs. (**a**) Tracheal mass located on the right dorsolateral aspect of the trachea and protruding into the lumen. (**b**) Surgical field following the resection of 10 tracheal rings. An endotracheal tube is visible within the distal trachea after being introduced through the tracheostomy site. (**c**) Resected tracheal specimen with 10 mm surgical margins (the caudal aspect is at the bottom of the image).

The dog was positioned in dorsal recumbency with the neck extended. A ventral midline skin incision was made from the caudal border of the cricoid cartilage and extended approximately 12 cm caudally, incorporating the temporary tracheostomy site. The paired sternothyroid muscles were separated along the midline raphe, and the trachea was exposed. The mass was located on the right dorsolateral aspect of the trachea and extended into the tracheal lumen. The carotid sheaths and recurrent laryngeal nerves were identified and carefully dissected bilaterally. The trachea was transected 20 mm caudal to the cricoid cartilage and one tracheal ring caudal to the tracheostomy site. A total of 10 tracheal rings were removed, corresponding to 24% of the total tracheal length. Cranial and caudal surgical margins of 10 mm were obtained (Figure 2).

**Figure 3:**
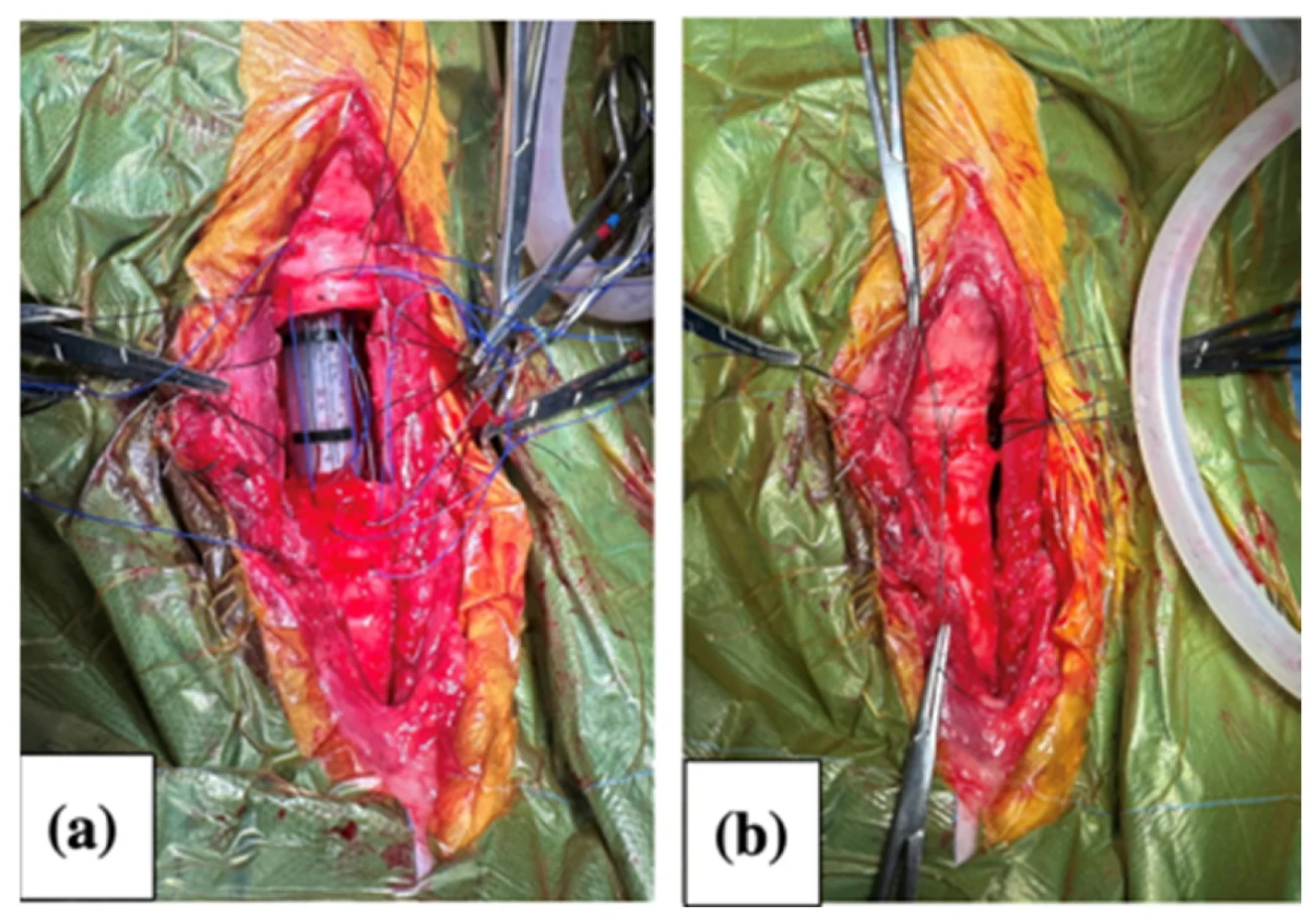
(**a**) Stay sutures were placed using 2-0 nylon, with the endotracheal tube repositioned orotracheally. (**b**) Completed end-to-end tracheal anastomosis (the caudal aspect is at the bottom of the image).

For the tracheal anastomosis, 2-0 nylon stay sutures were used. Three sutures were initially placed in the dorsal tracheal ligament, followed by additional interrupted sutures distributed circumferentially around the trachea, each engaging one cartilage ring cranial and one cartilage ring caudal to the resection site. Once all sutures had been placed, they were sequentially tied to complete the end-to-end anastomosis [21]. The remaining tissue layers were closed routinely using 3-0 Monosyn (Figure 3).

Anaesthesia was initially maintained using total intravenous anaesthesia with propofol, with the trachea intubated via the tracheostomy site. Once the end-to-end tracheal anastomosis was initiated, orotracheal intubation was performed, and maintenance was transitioned to partial intravenous anaesthesia, consisting of isoflurane delivered in 100% oxygen combined with a continuous rate infusion of dexmedetomidine (Table 1).

### 2.4. Histological Findings

The surgical specimen consisted of a tracheal segment containing 10 tracheal rings and a well-demarcated, firm, fleshy mass measuring 3 cm in diameter. The mass compressed the trachea and caused marked narrowing of the tracheal lumen (Figure 2c). The specimen was placed in formalin and submitted for histopathological examination.

**Figure 4:**
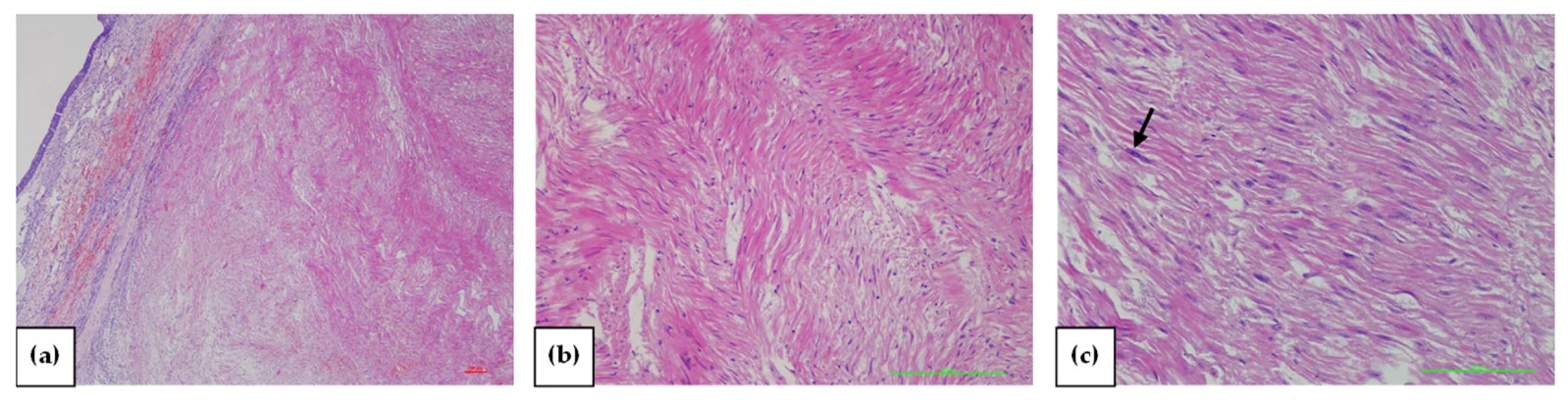
Photomicrographs of the tracheal leiomyoma. (**a**) Well-demarcated, unencapsulated, non-invasive neoplasm arising from the tracheal smooth muscle. Haematoxylin and eosin (H&E), ×40. (**b**) Neoplastic cells arranged in dense bundles separated by scant connective tissue, with indistinct cytoplasmic borders. H&E, ×200. (**c**) Elongated nuclei with rounded ends (arrow), giving them the characteristic cigar-shaped appearance. H&E, ×200.

Histopathological examination showed that the neoplasm arose from the tracheal smooth muscle. It was non-invasive, unencapsulated, and well-demarcated. The neoplastic cells were arranged in dense bundles separated by scant connective tissue and had indistinct cytoplasmic borders. The nuclei were elongated with rounded ends, giving them the characteristic cigar-shaped appearance. No mitotic figures were identified in the sections examined. The final diagnosis was tracheal leiomyoma (Figure 4). Immunohistochemical staining was not performed to confirm smooth muscle differentiation.

### 2.5. Outcome

The dog recovered without complications. Postoperative management included fluid therapy with lactated Ringer’s solution at a maintenance rate, together with multimodal analgesia consisting of methadone (0.1 mg/kg every 4 h) combined with metamizole (10 mg/kg every 12 h). Analgesia was progressively tapered according to serial pain assessments using the Glasgow Composite Measure Pain Scale.

Amoxicillin–clavulanic acid (20 mg/kg every 12 h) was administered as perioperative antimicrobial prophylaxis. Additional intravenous medications included maropitant as an antiemetic (1 mg/kg every 24 h) and dexamethasone for anti-inflammatory treatment (0.1 mg/kg every 24 h).

The surgical site was kept clean and dry throughout the hospitalisation period. The dog was discharged 48 h after surgery with no respiratory signs. At the follow-up examination 15 days after surgery, the dog showed no respiratory abnormalities, except for occasional mild coughing episodes when excited or nervous. Further follow-up was conducted by telephone, during which the owners reported a favourable clinical outcome. At the time of writing, two years after undergoing end-to-end tracheal anastomosis, the dog remains free of respiratory signs. The owners reported that her activity level had markedly increased and that she was able to run and play without respiratory distress or coughing.

## 3. Discussion

Primary tracheal neoplasms are uncommon in both veterinary and human medicine [23,24]. In humans, tracheal leiomyomas account for approximately 1% of all reported tracheal neoplasms, and more than 90% of reported tracheal tumours are malignant [16,25,26]. In dogs, tracheal neoplasms can be classified as cartilaginous or non-cartilaginous, with the latter being more frequent; tracheal leiomyoma is included within this group [27].

Consistent with previous reports, in which tracheal neoplasms are most commonly located in the cervical portion of the trachea and usually occur in middle-aged to geriatric dogs [13], the dog in the present case was diagnosed at 12 years of age, and the lesion was located at the level of the C3–C4 vertebral bodies.

The available literature on tracheal leiomyomas is limited. In human medicine, primary tracheal leiomyomas appear to occur more frequently in men, whereas pulmonary parenchymal leiomyomas have been reported more commonly in women. In this context, leiomyomatous lesions of the respiratory tract should be differentiated from benign metastasising leiomyoma, a rare entity described mainly in women with a history of uterine leiomyoma or previous uterine surgery. Therefore, assessment of reproductive and surgical history is relevant [28]. In the present case, the dog was a spayed female with no known history of uterine leiomyoma. In addition, CT was performed to rule out the presence of other masses, and no additional lesions or evidence of metastatic disease were identified. These findings, together with the solitary nature of the lesion, supported a primary tracheal origin of the neoplasm.

The main risk associated with this type of neoplasm does not necessarily lie in malignant biological behaviour, but rather in its ability to cause functional airway obstruction, which may severely compromise ventilation [11,28]. In this case, the clinical signs progressed gradually, with acute worsening that required temporary tracheostomy to stabilise the dog and allow planning of definitive surgical treatment.

The clinical signs were non-specific, as previously reported, and included respiratory abnormalities compatible with upper airway obstruction, such as inspiratory dyspnoea, tachypnoea, cyanotic episodes, and hoarseness [13,28,29]. Although oesophageal compression was observed on CT, the dog did not show dysphagia. On unenhanced CT, the mass showed a mean attenuation of 33 Hounsfield units (HU), consistent with soft-tissue attenuation. This value falls within the range reported for human tracheobronchial leiomyomas (25–46 HU). Although these tumours typically show homogeneous enhancement following contrast administration, the lesion in the present case showed minimal enhancement, with its mean attenuation increasing from 33 to only 34 HU [30].

Although intraluminal tracheal masses may be radiographically identified as well-defined lesions [5], in this case, thoracic and cervical radiographs did not reveal findings compatible with tracheal neoplasia. The intraluminal mass was not diagnosed until emergency bronchoscopy was performed because of worsening respiratory signs after left arytenoid lateralisation. This finding highlights the importance of considering intraluminal tracheal lesions in dogs with persistent obstructive signs, even when radiographic examination does not show evident abnormalities.

From an anatomical and surgical perspective, the trachea is part of the lower respiratory tract and, in mammals, consists of a sequence of C-shaped hyaline cartilage rings connected by annular ligaments and joined dorsally by the trachealis muscle [31]. In dogs, the trachea comprises approximately 35–46 tracheal rings (*cartilagines tracheales*) [32]. Although there is no definitive consensus regarding the maximum length of the trachea that can be safely resected in dogs, some studies suggest that up to 50% of the adult tracheal length may be removed [33]. However, resection of approximately 30% of the tracheal length has been reported to be associated with cranial displacement of the lungs [34]. In human medicine, resections exceeding 50% of the tracheal length usually require reconstructive techniques other than end-to-end anastomosis because of the tension generated at the suture line [35]. Conversely, studies in sheep have shown that up to 58% of the trachea can be resected without the development of complications [36].

The surgical technique used in this case was segmental tracheal resection with end-to-end anastomosis, as previously described in the veterinary surgical literature [21]. In the present dog, the resection represented 24% of the total tracheal length. A more extensive resection was not required, as adequate macroscopic surgical margins of 10 mm were obtained both cranially and caudally. This percentage is within the limits considered safe in the literature, which probably contributed to reducing the risk of complications associated with anastomotic tension [37]. Accordingly, it was not necessary to maintain the neck in flexion during the postoperative period or to use postoperative tension-relieving sutures, such as chin-to-chest sutures. A 2-0 non-absorbable monofilament suture (nylon) was used to construct the anastomosis. However, finer absorbable monofilament suture material is more commonly described in the scientific literature [21,34].

Regarding surgical margins, specific evidence for canine tracheal tumours is limited. However, in some malignant tracheal neoplasms, such as squamous cell carcinoma, margins greater than 5 mm have been described as sufficient [20]. In this case, because the tumour was a leiomyoma and complete resection was achieved, the prognosis was considered favourable [38]. This was supported by the clinical outcome, as the dog remained free of respiratory signs and showed no evidence of recurrence during a two-year follow-up period. Owing to the broad base of attachment and the extent of the lesion, endoscopic excision under bronchoscopic guidance was not considered appropriate.

Although the surgical technique of tracheal resection and anastomosis is well-described in veterinary medicine, the available information on anaesthetic management during these procedures remains limited [39,40,41]. In the present case, the dog did not require retrograde intubation or the use of a supraglottic airway device. Initially, ventilation was performed through the previously placed temporary tracheostomy, which allowed safe airway control during the initial phase of the procedure. Once the end-to-end anastomosis had been initiated, the dog was intubated orotracheally, allowing completion of the tracheal reconstruction without significant interference with the surgical field.

In human medicine, although mortality associated with tracheal resection and reconstruction in specialised centres is low, approximately 1–2%, the complication rate may reach 18.2% [42]. In the present case, after tracheal resection and end-to-end anastomosis, the dog did not develop postoperative complications. Placement of a cervical guardian suture to limit neck extension and reduce tension on the anastomosis was not required. Likewise, no relevant anaesthetic complications, such as episodes of hypoxaemia, which have previously been described in some reports of anaesthetic management of tracheal surgery in small animals, were observed [39]. Regarding LP, the most commonly reported cause is idiopathic, although acquired causes such as bite trauma or mediastinal masses have also been described [43]. Less frequent causes include mineralisation of the surrounding muscles [44] and muscular pseudotumours [45]. In the present case, LP was suspected to be secondary to adhesion of the tracheal tumour to the recurrent laryngeal nerve, although a definitive causal relationship could not be confirmed. Arytenoid lateralisation contributed to improved airflow following removal of the tracheal mass. The mild change in bark tone noted at admission was attributed to LP [43] and persisted throughout long-term follow-up. Its persistence after surgery may have been associated with left arytenoid lateralisation, as maintaining the arytenoid cartilage in an abducted position may affect vocal fold tension and vibration. Manipulation of the vagus nerve or its recurrent laryngeal branch during dissection of the adherent mass may also have contributed. Although changes in airway resonance following segmental tracheal resection cannot be ruled out, this was considered a less likely explanation. Consequently, the change in bark tone could not be attributed with certainty to a single cause.

Overall, this case highlights the importance of including intraluminal tracheal neoplasms in the differential diagnosis of dogs with persistent obstructive airway signs, even when radiographic findings are non-specific. Bronchoscopy and CT were essential for diagnosis and surgical planning. Segmental tracheal resection with end-to-end anastomosis allowed complete excision of the lesion, with a favourable clinical outcome and no relevant perioperative complications.

## 4. Conclusions

Tracheal leiomyomas are rare neoplasms in both veterinary and human medicine. Although they may cause marked respiratory signs due to functional airway obstruction, complete surgical resection of the mass may be curative. However, the management of these lesions requires close coordination between the surgical and anaesthetic teams, particularly to ensure adequate airway control throughout the procedure.

At the time of writing, two years had elapsed since surgery, with no recurrence of respiratory signs. The owners reported only a mild change in the tone of the bark, similar to that noted at the onset of the clinical signs.

This case highlights the importance of a multidisciplinary approach to rare or complex conditions, such as tracheal leiomyoma, and demonstrates that appropriate diagnostic, surgical, and anaesthetic planning can lead to favourable long-term clinical outcomes.

## Figures and Tables

**Table 1:** Perioperative anaesthetic, analgesic, antimicrobial, and fluid therapy administered to the dog.

Perioperative Phase	Agent	Dose and Route	Recommended Dose Range *	Administration Details
Antimicrobial prophylaxis	Ampicillin (Gobemicina^®^)	11 mg/kg IV	10–20 mg/kg IV	Administered 20 min before skin incision and repeated every 90 min intraoperatively
Premedication	Methadone (Semfortan^®^)	0.3 mg/kg IM	0.1–0.5 mg/kg IM	Single administration
Anaesthetic induction	Propofol (Propomitor^®^)	1 mg/kg IV	2–6 mg/kg IV	Administered slowly and titrated to effect
Intraoperative analgesia	Methadone (Semfortan^®^)	0.1 mg/kg IV	0.1–0.3 mg/kg IV	Diluted and administered slowly 3 h after premedication
Anaesthetic maintenance	Propofol (Propomitor^®^)	0.25 mg/kg/min IV	0.1–0.4 mg/kg/min IV	Infusion rate adjusted according to anaesthetic requirements.
Anaesthetic maintenance	Dexmedetomidine (Sedadex^®^)	1 µg/kg/h IV	1–2 µg/kg/h IV	Administered as a CRI, with the final solution infusion at 1 mL/kg/h
Anaesthetic maintenance	Isoflurane (Isoflurin^®^)	0.1–0.2% in oxygen, by inhalational	1.5–2.5% in oxygen, by inhalational	Adjusted according to anaesthetic depth
Maintenance fluid therapy	Lactated Ringer’s solution	5 mL/kg/h IV	3–5 mL/kg/h IV	Adjusted according to the dog’s requirements
Fluid bolus	Lactated Ringer’s solution	5 mL/kg IV over 15 min	3–5 mL/kg IV over 15–20 min	Administered according to haemodynamic requirements and the dog’s response

* Recommended dose and concentration ranges were obtained from the BSAVA Manual of Canine and Feline Anaesthesia and Analgesia [22]. Abbreviations: CRI, continuous rate infusion; IM, intramuscular; IV, intravenous.

## Data Availability

All relevant clinical data supporting the findings of this case report are included in the article. Additional information is available from the corresponding author upon reasonable request, subject to applicable privacy and ethical considerations.

## Abbreviations

The following abbreviations are used in this manuscript:

LP	Laryngeal paralysis
CT	Computed tomography
HU	Hounsfield units
